# Supplementary material for: Kinesiophobia among health professionals’ interventions: a scoping review
Source: PeerJ. 2024 Aug 22;12:e17935. doi: 10.7717/peerj.17935 (PMC11344999; doi:10.7717/peerj.17935)
Supplement: Appendix S1 [file peerj-12-17935-s001.docx]

**Appendix 1: Boolean operators used throughout the databases.**

| **PubMed**  ("Pain-Related Fear"[Title/Abstract] OR "avoidance behavio*"[Title/Abstract] OR "pain belief*"[Title/Abstract] OR "fear of movement"[Title/Abstract] OR kinesiophob*[Title/Abstract] OR "fear avoidance"[Title/Abstract] OR "anticipation of pain"[Title/Abstract] OR ("Fear"[Mesh:NoExp] AND "Movement"[Mesh:NoExp]) AND ("Nurses"[Mesh:NoExp] OR "Physical Therapists"[Mesh] OR "Physicians, Primary Care"[Mesh] OR "Physicians"[Mesh:NoExp] OR "Physicians, Family"[Mesh] OR "Rheumatologists"[Mesh] OR "physical therapist*"[Title/Abstract] OR Physiotherapist*[Title/Abstract] OR kinesiotherapist*[Title/Abstract] OR clinician*[Title/Abstract] OR "health professional*"[Title/Abstract] OR "health personnel*"[Title/Abstract] OR practitioner*[Title/Abstract] OR chiropractor*[Title/Abstract] OR physician*[Title/Abstract] OR nurse*[Title/Abstract] OR "health care provider*"[Title/Abstract] OR Rheumatologist*[Title/Abstract]) |
| --- |
| **Sportdiscus**  (SU ("NURSES" OR "PHYSICIANS" OR "PHYSICAL therapists" ) OR TI ("physical therapist*" OR Physiotherapist* OR kinesiotherapist* OR clinician* OR "health professional*" OR "health personnel*" OR practitioner* OR chiropractor* OR physician* OR nurse* OR "health care provider*" OR Rheumatologist* ) OR AB ("physical therapist*" OR Physiotherapist* OR kinesiotherapist* OR clinician* OR "health professional*" OR "health personnel*" OR practitioner* OR chiropractor* OR physician* OR nurse* OR "health care provider*" OR Rheumatologist* )) AND ((SU ("avoidance (psychology)" OR ("body movement" AND "fear") )) OR TI ("Pain-Related Fear" OR "avoidance behavio*" OR "pain belief*" OR "fear of movement" OR kinesiophob* OR "fear avoidance" OR "anticipation of pain") OR AB ("Pain-Related Fear" OR "avoidance behavio*" OR "pain belief*" OR "fear of movement" OR kinesiophob* OR "fear avoidance" OR "anticipation of pain")) OR nurse* OR "health care provider*") ) ) |
| **CINAHL**  ( ( ((MH "Fear") AND (MH "Movement")) OR (MH "Kinesiophobia") ) OR TI ( (" Pain-Related Fear" OR "avoidance behavio*" OR "pain belief*" OR "fear of movement" OR kinesiophob* OR "fear avoidance" OR "avoidance (psychology)" OR "anticipation of pain") ) OR AB ( (" Pain-Related Fear" OR "avoidance behavio*" OR "pain belief*" OR "fear of movement" OR kinesiophob* OR "fear avoidance" OR "avoidance (psychology)" OR "anticipation of pain") ) ) AND ( ( (MH "Health Personnel") OR (MH "Physical Therapists") OR (MH "Chiropractors") OR (MH "Nurses") OR (MH "Physicians") OR (MH "Rheumatologists") OR (MH "Physicians, Family") OR OR (MH "Health Personnel") OR (MH "Physical Therapists") OR (MH "Chiropractors") OR (MH "Nurses") OR (MH "Physicians") OR (MH "Rheumatologists") OR (MH "Physicians, Family") ) OR TI ( ("physical therapist*" OR Physiotherapist* OR kinesiotherapist* OR clinician* OR "health professional*" OR "health personnel*" OR practitioner* OR chiropractor* OR physician* OR nurse* OR "health care provider*") ) OR AB ( ("physical therapist*" OR Physiotherapist* OR kinesiotherapist* OR clinician* OR "health professional*" OR "health personnel*" OR practitioner* OR chiropractor* OR physician* OR nurse* OR "health care provider*") ) ) |
